# Effects of Phenolic Compounds of Fermented Thai Indigenous Plants on Oxidative Stress in Streptozotocin-Induced Diabetic Rats

**DOI:** 10.1155/2011/749307

**Published:** 2011-03-08

**Authors:** Chaiyavat Chaiyasut, Winthana Kusirisin, Narissara Lailerd, Peerasak Lerttrakarnnon, Maitree Suttajit, Somdet Srichairatanakool

**Affiliations:** ^1^Department of Pharmaceutical Science, Faculty of Pharmacy, Chiang Mai University, Chiang Mai 50200, Thailand; ^2^Department of Family Medicine, Faculty of Medicine, Chiang Mai University, Chiang Mai 50200, Thailand; ^3^Department of Physiology, Faculty of Medicine, Chiang Mai University, Chiang Mai 50200, Thailand; ^4^Faculty of Science and Technology, Phayao University, Phayao Province 56000, Thailand; ^5^Department of Biochemistry, Faculty of Medicine, Chiang Mai University, Chiang Mai 50200, Thailand

## Abstract

We investigated the effects of antioxidant activity of fermentation product (FP) of five Thai indigenous products on oxidative stress in Wistar rats with streptozotocin (STZ)-induced diabetes type II. The rats were fed with placebo and with the FP (2 and 6 mL/kg body weight/day) for 6 weeks. Rutin, pyrogallol and gallic acid were main compounds found in the FP. Plasma glucose levels in diabetic rats receiving the higher dose of the FP increased less when compared to the diabetic control group as well as the group receiving the lower FP dose (13.1%, 29%, and 21.1%), respectively. A significant dose-dependent decrease in plasma levels of thiobarbituric acid reactive substance (*P* < .05) was observed. In addition, the doses of 2 and 6 mL FP/kg/day decreased the levels of erythrocyte ROS in diabetic rats during the experiment, but no difference was observed when compared to the untreated diabetic rat group. Results imply that FP decreased the diabetes-associated oxidative stress to a large extent through the inhibition of lipid peroxidation. The FP also improved the abnormal glucose metabolism slightly but the difference was not statistically significant. Thus, FP may be a potential therapeutic agent by reducing injury caused by oxidative stress associated with diabetes.

## 1. Introduction

Diabetic mellitus (DM) comprises a group of common metabolic disorders that share the phenotype of hyperglycemia which affects the metabolism of carbohydrates, lipids and proteins. Prolonged periods of hyperglycemia lead to glucose degradation within living tissues, causing accumulation of glucose within organ cells, which can result in complications of DM such as coronary heart disease, retinopathy, nephropathies, and neuropathies [1–3]. In 2007, the International Diabetes Federation reported that the worldwide prevalence of DM has risen dramatically. It is estimated that by 2025 the prevalence of DM worldwide will rise from 246 million to 380 million people. The etiology and pathophysiology of have been a topic of much interest in recent years especially the relationship between free radicals produced by the metabolism of proteins, DNA, and lipids in living organisms and the increase in oxidative reactions which result in molecular and tissue damage in the body. 

The cellular toxicity caused by overproduction of free radicals is called oxidative stress. Therefore, free radical production resulting in oxidative stress is a popular theory that explains the etiology and pathophysiology of the biological effects of DM, especially in regard to cell damage, cellular degeneration, and subsequent complications. Reports both in vitro and in vivo have demonstrated that hyperglycemia causes an increase of free radical in cells, which leads to acute toxicity and molecular destruction, affecting the cellular mediators. These conditions lead to insulin resistance, beta cell dysfunction, and impaired insulin secretion resulting in the complications of type II DM. In the literatures, antioxidants enable to decrease or inhibit oxidation can be found naturally or synthesized from fermentation of *Rhus hirta*, *Quercus alba,* and *Cornus stolonifera* [1, 4–7]. Natural antioxidants such as polyphenolic compounds and their effects against free-radical scavenging and oxidative stress have been studied [8–12]. Biologically fermented plant products appear as a clear brown liquid with a sour taste due to the fermentation of plants, herbs, vegetables, or fruits with sugar in a closed environment with lactic producing bacteria or probiotic bacteria. A previous study indicated that they are rich in antioxidants with antioxidative activity similar to butylated hydroxyanisole and green tea [13]. Furthermore, studies indicate that the antioxidative activity increased upon fermentation, which dissolves the ingredients and bacteria to release useful chemicals and phytochemicals in the process. During *Lactobacillus* fermentation organic acids are formed and accumulated, possibly leading to protein hydrolysis and solubilisation of antioxidant ferulic acid from cell wall plant materials [14–16]. Fermentation of grain food with *Aspergillus oryzae* possesses strong antioxidant and free-radicals scavenging activities [17]. Antioxidative and anti-inflammatory activities of the biologically fermented plant products can be utilized alternatively for improving health as well as treatment for patients with such diseases as HIV and cancer [18]. 

Biologically fermented plants locally made in Thailand and other countries are consumed to improve health. In 2004, a report was presented in a meeting on standards and safety of biologically fermented plant products for consumption hosted by the National Science and Technology Development Agency in Thailand. The report stated random interviews of 235 biologically fermented plant product consumers nationwide who 10.2 percent drank the product to treat diabetic mellitus (DM). Nevertheless, very little scientific evidence on the impact of health in vitro and vivo on diabetic patients exist. Thus, this study aimed to assess the effect of selected Thai biologically fermented plant products on oxidative stress in streptozotocin- (STZ-) induced diabetes type II Wistar rats. Levels of oxidative stress makers including plasma TBARS, superoxide anion (O_2_^•−^) and nitric oxide (NO), and erythrocyte radicals were measured.

## 2. Materials and Methods

### 2.1. Chemicals and Plant Product

Streptozotocin (STZ), malondialdehyde (MDA), 2,2′-azino-*bis*(3-ethylbenzthiazoline-6-sulphonic acid) (ABTS), 1,1′,3,3′-tetramethoxypropane, thiobarbituric acid (TBA), Folin-Ciocalteu's reagent, rutin, gallic acid, pyrogallol, catechin, and caffeic acid were purchased from Sigma-Aldrich Chemical Company (St. Louis, MO, USA). 2′,7′-Dichlorofluorescein diacetate (DCFH-DA) was obtained from Molecular Probes (Eugene, OR, USA). *β*-Nicotinamide adenine dinucleotide, reduced disodium salt (NADH), 2H-(tetrazolium,3,3′-(3,3′-dimethoxy (1,1′-biphenyl)-4,4′-diyl) *bis*(4-nitrophenyl)-5-(phenyl) dichloride or nitroblue tetrazolium chloride (NBT), ethylenediaminetetraacetic acid (EDTA) disodium salt, and phenazine methosulphate (PMS) were purchased from Fluka Chemicals (Buchs Switzerland). Ultrapure grade water (Milli-Q water) was from Millipore Corporate (Billerica, MA, USA). Ethyl acetate (AR grade) and methanol (HPLC grade) were purchased from Mallinckrodt Company (Mallinckrodt J.T. Baker, Inc. Phillipburg, NJ, USA). Other chemicals were analytical reagent or the highest pure grade.

### 2.2. Preparation of Starter Culture Inoculum

Lactic acid producing bacteria (*Lactobacillus casei*) was used as starter culture and identified by using biochemical tests and API 50CH carbohydrate fermentation strip test (bioMérieux SA Inc., Marcy l′Etoile, France). Preculture plates of the isolate were prepared by streaking a loopful of stock starter culture on Difco Lactobacilli MRS Agar plate (Difco, Detriot, MI USA) and incubated at 37 ± 1°C in 6.1% CO_2_ for 48 hours. Single colony was then inoculated into Difco Lactobacilli MRS Broth (Difco, Detriot, MI USA) and incubated at 37 ± 1°C in 6.1% CO_2_ for 10–15 hours. The broth culture (approximately 10^9^ CFU/mL) was transferred to the fermented medicinal plant juice (FMPJ) (10%, v/v) and incubated at 37 ± 1°C for 18–25 hours.

### 2.3. Preparation of Fermentation Product

Fermentation product of five Thai indigenous plants was prepared at the Research and Development of Health Product Unit, Department of Pharmaceutical Science, Faculty of Pharmacy, Chiang Mai University, Thailand. The plants including Malacea tree* (Phyllanthus emblica* Linn., PE), Indian mulberry (*Morinda citrifolia* Linn., MC), Heart leaf (*Houttuynia cordata *Thunb., HR), Myrobalan (*Terminalia chebula *Retz., TC), and Krachai-Dam (*Kaempferia parviflora* Wall., KP) were used in this study. In preparation, the plants were cut into small pieces and crushed with the crusher. The crushed plants (3.47 kg each) were mixed with cane sugar (1.16 kg) and reverse osmosis water (11.57 kg), then inoculated with the starter culture (1.8 kg) containing potassium metabisulfite (250 mg/l). The mixture was contained in an air locked rubber polypropylene tank (18.9 liter capacity), fermented separately in an incubation room at 30 ± 2°C for 1 month and filtered. The fermentative juices of the PE, TC, KP, MC, and HC (10 : 10 : 5 : 20 : 55, v/v/v/v/v) were mixed together “called fermentation product (FP)” and used in the experiments. The FP was further determined antioxidant activity and identified phenolic compounds as described below.

### 2.4. Preparation of Sample Solution

The isolation of phenolics from the FP was performed by solid phase extraction (SPE) [19]. Briefly, the SPE column (C18 cartridge, 3-mL capacity, 5% carbon load, Whatman, Maidstone, Kent, UK) was washed with ethyl acetate (3 mL), methanol (3 mL) and Milli Q water (5 mL), respectively. The FP (1.0 mL) was loaded onto the column and washed three times with deionized water. Compounds retained in the column were eluted with ethyl acetate (3.0 mL). The eluate was evaporated at room temperature and the residue was reconstituted with methanol (1.0 mL). Solutions of reference standards such as rutin, gallic acid, pyrogallol, catechin, and caffeic acid were prepared in methanol. The solutions were filtered through a 0.45-*μ*m syringe filter before use.

### 2.5. Identification of FP Components by HPLC and GC-MS Techniques

The analytical Agilent 1100 Series HPLC system (Agilent Technologies, Santa Clara, CA, USA) comprises quaternary high pressure pumps, thermoregulated autosampler, photodiode-array detector, a modular HP ChemStation Software installed in a computer system. Mobile-phase solvent consisting of water/ 0.4% acetic acid/methanol/acetronitrile (70 : 20 : 5 : 5, v/v/v/v) was filtered (0.45 *μ*m membrane, polysulphone type) and ultrasonically degassed before use. Firstly, the column was calibrated using standard rutin, pyrogallol, gallic acid, catechin, and caffeic acid to determine their specific retention times (RT). The sample and the reference standards (6 *μ*L) were applied to the Hypersil column (ODS type, 250 mm × 4 mm id., 5 *μ*m, Thermo Fisher Scientific Inc., Waltham, MA, USA) temperature-controlled at 25°C and eluted with the mobile phase at a flow rate of 0.7 mL/min. Eluents were monitored with the PDA detector at the wavelengths of 200, 280, and 360 nm. Data were recorded and analyzed with the ChemStation Software. 

Active ingredients of the FP were also analyzed by using a GC-MS technique at the Science and Technology Service Center, Chiang Mai University, Thailand. A gas chromatography machine (Agilent Technologies, Inc., Santa Clara, CA, USA) used advanced electronic pneumatics control (EPC) and extremely accurate temperature control. The system was included a column (HP-5MS Agilent, 5%-phenyl-methylpolysiloxane, 30 m × 0.25 mm i.d., 0.25 *μ*m film thick) and conditioned with inlet of 270°C, 1.0-*μ*L split ratio (10 : 1), oven temperature of 50°C (12°C/min) → 260°C (42.5 min), a flow rat of helium gas carrier at 1.0 min/min. A mass spectrometer (Agilent 5975 Series MSD, Triple-Axis HED-EM detector programmable temperature up to 350°C) was online connected to the GC machine. Data of analysis were acquired using ChemStation program. Mass spectra of analyzed samples were compared with those of standard compounds as stored in a database library.

### 2.6. ABTS Radical Decolorizing Assay

The method used is based on the ability of antioxidative compounds to quench the ABTS radical cation (ABTS^●+^), a blue-green chromophore with characteristic absorption at 734 nm, compared with the ascorbic acid, a water-soluble vitamin C analog [20, 21]. A stable stock solution of ABTS^●+^ was produced by reacting aqueous solution of ABTS (7 mM) with potassium persulfate solution (a final concentration of 2.45 mM) and allowed the mixture to stand in a dark at room temperature for 12–16 hours before use. The solution was diluted with ethanol to obtain an absorbance of 0.70 ± 0.02 unit at 734 nm. The FP was dissolved in ethanol. An aliquot of each sample (20 *μ*L) in ethanolic solution was added into 2.0 mL of ABTS^●+^ solution, the absorbance was monitored for 3 min at 734 nm, verified by a UV/VIS spectrophometer (Jasco model 7800). The antioxidant activity was expressed as mg ascorbic acid equivalents (mg AE/mL of FP).

### 2.7. Quantification of Phenolics Content

Content of total phenolics in the FP was determined by Folin-Ciocalteu reagent [22]. A dilute extract of the FP (0.5 mL of 1 : 10 g/mL) or gallic acid (standard phenolic compound) was mixed with Folin-Ciocalteu reagent (5 mL, 1 : 10 diluted with distilled water) and aqueous Na_2_CO_3_ (4 mL, 1 M). The mixtures were allowed to stand for 15 min and the total phenolics content was determined by colorimetry at 765 nm. The standard curve was prepared using gallic acid in methanol/water (50 : 50, *v*/*v*) and expressed as gallic acid equivalents (mg GAE/mL of FP).

### 2.8. Analysis of Flavonoids Content

The flavonoids content was determined by the aluminum chloride colorimetric method [23]. The FP sample (0.5 mL of 1 : 10 g/mL in methanol was separately mixed with 1.5 mL of methanol, 0.1 mL of 10% aluminum chloride, 0.1 mL of 1 M potassium acetate and 2.8 mL of distilled water. It remained at room temperature for 30 min; the absorbance was measured at 415 nm. The standard curve was prepared by preparing various concentrations of quercetin in methanol and expressed as quercetin equivalents (mg QE/mL of FP). Quercetin is the major flavonoids occurring ubiquitously in foods of plant origin and has been frequently used as a model compound showing the antioxidant property [24].

### 2.9. Determination of Hydrolysable Tannins Content

Phenolics are secondary plant metabolites, which tannins is the group of the phenolics and potential biological antioxidant. Folin-Denis assay is the most widely used method for quantification of total phenolics in plant materials and beverages. Content of hydrolysable tannins (such as gallotannins and ellagitannins) in the FP was determined by a modified method of [25]. The FP sample (0.1 mL) was mixed with 0.5 mL Folin-Denis reagent followed by 1 mL of Na_2_CO_3_ (0.5%) solution and distilled water (up to 5 mL). The optical density (OD) was measured at 775 nm within 30 min of the reaction against the reagent blank. Results were expressed as mg GAE/mL of FP.

### 2.10. Animal Study

Male Wistar rats (180–200 g) were purchased from the National Animal Laboratory Center, Mahidol University at Salaya Campus, Bangkok, Thailand. The rats were housed in stainless-steel cages, with constantly- controlled room temperature (23 ± 2°C), humidity temperature (65 ± 10%), and 12/12 h light/dark cycle. They were fed a standard chow diet containing 12% moisture, 24% crude protein, 4.5% fat, 5% fiber, 3040 kcal/kg metabolic energy, 1% Ca, 0.9% P, 0.2% Na, 1.17% K, 0.23% Mg, 171 ppm Mn, 22 ppm Cu, 100 ppm Zn, 180 ppm Fe, 1.82 ppm Co, 0.1% Se, 20000 IU/kg vitamin A, 4000 IU/kg vitamin D, 100 mg/kg vitamin E, 5 mg/kg vitamin K, 20 mg/kg vitamin B1, 20 mg/kg vitamin B2, 20 mg/kg vitamin B6, 20 mg/kg vitamin B12, 100 mg/kg niacin, 6 mg/kg folic acid, 0.4 mg/kg biotin, and 1500 mg/kg choline chloride. Sterile deionized water was filled in a clean glass bottle and given ad libitum. All procedures were approved by the Ethics Committee for the use of experimental animals in the Faculty of Medicine, Chiang Mai University (Protocol no. 10/2549).

### 2.11. Experimental Design

All rats were acclimatized for a one-week period, then weight-matched into normal and diabetes type II control groups. A single dose of STZ 45 mg/kg body weight (BW) was injected intraperitoneally (IP) to induce diabetes in one [26] and the other received vehicle only. One week after the STZ treatment, blood samples were taken from tail vein and plasma glucose levels were measured. Rats with fasting plasma glucose levels above 250 mg/dL were regarded as having diabetes. The rats were randomly divided into four groups and FP was administrated to the experiment group by oral gavage feeding once a day as follows.

Group 1: normal control rats (*n* = 9) given vehicle only.
Group 2: the diabetic control rats (*n* = 13) given vehicle only.
Group 3: the diabetic rats (*n* = 11) treated with FP at a dose of 2 mL/kg BW.
Group 4: the diabetic rats (*n* = 12) treated with FP at a dose of 6 mL/kg BW.

Their fasting (8–12 hrs) bloods were collected every 2 weeks for analysis. The rats were treated for 6 weeks and sacrificed.

### 2.12. Measurement of Plasma Glucose Concentration

The blood was collected in a sodium fluoride/oxalate tube and spun in a refrigerated centrifuge (Hettich MIKRO 22R, Andreas Hettich GmbH & Co. KG, Tuttlingen, Germany) with a 35° angle rotor at 3500 rpm (1123 g), 4°C for 15 min. Plasma glucose concentration was determined with glucose oxidase/peroxidase method [27] using a commercial assay kit supplied by BIOTECHNICAL Company, Bangkok, Thailand. The assay procedure was followed according to the manufacturer instruction.

### 2.13. Determination of Plasma TBARS

TBARS method was used to evaluate lipid peroxidation [28] and slightly modified. Briefly, blood samples of healthy and diabetic rats were collected in heparinized tube and spun in the refrigerated Hettich centrifuge at 1123 g, 4°C for 15 min. The plasma was removed to measure for lipid peroxidation by TBARS assay. Forty microlitres of 0.2% butylated hydroxytoluene were added into plasma sample (0.375 mL) and mixed gently. The mixture was divided into three equal aliquots (one was used as a sample blank, and the others were used as a duplicate). After 750 *μ*L of phosphoric acid (0.44 M H_3_PO_4_) were added to each tube, 250 *μ*L of 0.6% (w/v) TBA reagent were added to both assay tubes and 250 *μ*l of deionized water were added to the blank tube. All mixtures were incubated at 90°C for 30 min, cooled down at room temperature, and the absorbance was read at 532 nm against the reagent blank. Data obtained from three independent experiments of the duplicate were expressed as MDA equivalents (*μ*M) using 1,1,3,3-tetramethoxypropane as a reference standard.

### 2.14. Fluorescent-Labeled Flow Cytometry of Erythrocyte Reactive Oxygen Species

Red blood cells (RBC) oxidative stress was analyzed by the flow cytometry [29]. Briefly, whole blood (5 *μ*L) was diluted with phosphate buffered saline (PBS) to reach 1 × 10^6^ RBC/mL. Then, 20 *μ*L of 0.4 mM DCFH-DA (dissolved in methanol) were added and incubated for 15 min at room temperature. The cell suspension (250 *μ*L) was spun in the refrigerated Hettich centrifuge at 3502 g, 10°C for 10 min and the excess dye was removed. The RBC pellet was washed three times with PBS and resuspended in 500 *μ*L of PBS. The content of reactive oxygen species (ROS) was measured with a flow cytometer (BD FACSCalibur, BD Biosciences, Mississauga, ON, Canada) using saline as the sheath fluid, which cell count was set at 10,000 cells. The fluorescence intensity (FI) was measured at an excitation wavelength of 350 nm and an emission wavelength of 450 nm. BD CellQuest v3.3 program software provided user-defined calculation, reagent and sample-specific gating, and overlays.

### 2.15. Superoxide Anion (O_2_^•−^) Scavenging Activity Assay

Superoxide level was measured using a colorimetric method [30]. The O_2_^•−^ scavenging activity was determined by measuring the decrease in ratio of the reduction of NBT. The plasma samples were added to the reaction buffer (50 mM PBS pH 7.4, 125 *μ*M EDTA, 62 *μ*M NBT, and 98 *μ*M NADH) containing 33 *μ*M PMS. After incubation at 37°C for 30 min, the absorbance was measured at 560 nm, as an index of NBT reduction, using a 96-well microplate reader (Multimode detector, Beckman Coulter, Inc., Fullerton, CA, USA).

### 2.16. Nitric Oxide Radical (NO^•^) Inhibition Assay

Nitric oxide radical inhibition was estimated via the nitrite and nitrate levels following the method [31]. Briefly, nitrate in plasma was converted to nitrite by incubation with nitrate reductase in PBS (pH 7.4). Nitrite and nitrate levels were measured by a microplate assay method based on the Griess diazotization reaction. Griess reagent (Promega Corporation, Madison, MA, USA) contained 0.2% *N*-1-naphthylenediamine dihydrochloride and 2% sulphanilamide in 5% phosphoric acid. A pink colored chromophore is formed in diffuse light. The absorbance of these solutions was measured at 520 nm against the corresponding blank solutions. Sodium nitrite was used as a standard.

### 2.17. Statistical Analysis

Data were expressed as means ± standard error of means (SEM). They were analyzed by the difference between groups by one-way analysis of variance (ANOVA), using Dunnett's test and those at *P* < .05 were considered to be statistically significant.

## 3. Results and Discussion

### 3.1. HPLC/UV Absorption and GC/MS Analyses of Phenolic Compounds

Our recent study has shown that the PE, TC, MC, KP, and HC constituted phenolic acids (2535 ± 68, 2275 ± 436, 50 ± 8, 100 ± 8 and 295 ± 22 mg GAE/g, resp.), flavonoids (50 ± 1, 99 ± 6, 68 ± 1, 104 ± 1 and 191 ± 2 mg QE/g, resp.), and tannins (0.20 ± 0.01, 0.15 ± 0.01, 0.53 ± 0.02, 0.85 ± 0.01 and 0.80 ± 0.01 mg tannic acid equivalent (TAE)/g, resp.) [32]. In this study, one milliliter of the FP was found to have different levels of subclasses of phenolic compounds as following: phenolics 27.3 ± 1.1 mg GAE, total flavonoid 2.24 ± 0.02 mg QE, and hydrolyzable tannins 0.051 ± 0.002 mg. It also had an antioxidant activity index of 31.31 mg GAE/mL of the FP (Table 1). 

**Figure 1:**
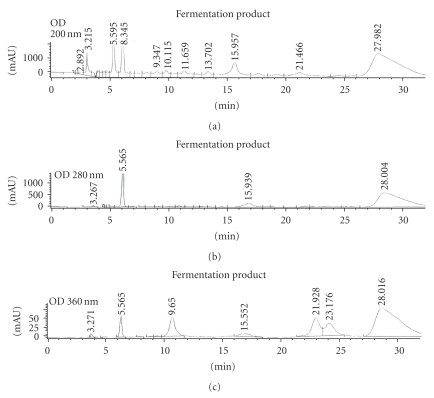
HPLC/UV absorption analysis of the FP. Eluents of the FP were compared with the phenolic standards: rutin, gallic acid, pyrogallol, catechin, and caffeic acid as previously mentioned in the Method Section.

Bramati and colleagues applied the HPLC/UV method to the quantitative characterization of major flavonoids in unfermented and fermented indigenous plants and found rutin (1.69 ± 0.14 mg/g) was one of major components [33]. Result of HPLC/200-nm detection demonstrated appearance of the rutin, gallic acid, pyrogallol, catechin, and caffeic acid peaks 3.2, 5.5, 6.3, 22.7, and 30.4 min, respectively, (Figure 1). In HPLC profile of the FP we found five main compounds including rutin (RT = 3.21 min), gallic acid (5.56 min), pyrogallol (6.34 min), and two other phenolics (RT = 15.96 and 27.98 min); however, caffeic acid was not detected. When amounts of these compounds were estimated from the corresponding peak areas, they were 2.76% rutin, 5.58% gallic acid, 6.60% pyrogallol, 6.03% catechin, 9.70% unidentified compound I (RT 15.96 min), and 38.83% unidentified compound II (RT 27.98 min). Gallic acid and the unidentified compound II exhibited strong UV absorption at 280 nm (11.07 and 74.35% peak area, resp.). The observation of 360 nm absorbance compounds possibly identified flavonoids group present in the FP. 

**Figure 2:**
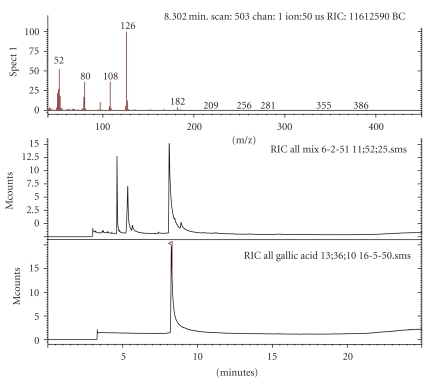
GC/MS identification of active ingredients of the FP (top) and of standard gallic acid (bottom).

GC/MS method was used to profile polyphenolic compounds in microbial fermentation products [34]. Here, GC/MS analysis demonstrates the gallic acid was one of three main components present in the FP (Figure 2). Pyrogallol was measured as a metabolite of gallotannin whereas gallic acid was formed by hydrolysis of the gallotannins or tannic acid. Tannins are unfeasible for GC analysis due to their large molecular weight, polarity and thermal instability. Nonetheless, pyrolysis-GC/MS can rapidly analyze the degradation products and characterize the original samples. With the chromatographic analysis fermentation of five Thai indigenous plants possibly produced large amounts of gallic acid, pyrogallol, catechin, and small amount of condensed (polymeric) tannins.

### 3.2. Effect of FP on Body Weight

**Figure 3:**
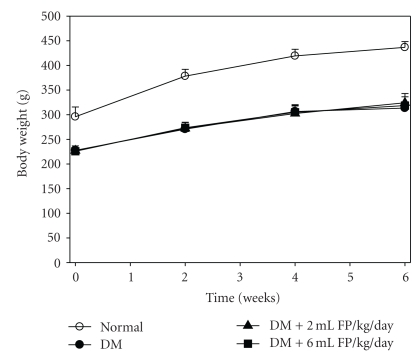
Effect of the FP on body weight of STZ-induced diabetic rats. Data are expressed as mean ± SEM. ^#^ *P* < .05 when compared with the normal control group.

Result in Figure 3 shows the effects of FP on the changes in body weight of rats after the 6 week experimental period. The average body weight of the diabetic control group was initially 170.4 ± 3.0 g and progressively increased to 313.5 ± 12.5 g (increase 37.7%) by the 6th week. The weight gain in the normal control group during the experimental period was 47.6%. The increase in body weight of all diabetic groups was significantly lower than the normal rats (*P* < .05) at week 0 and at the 2nd–6th week (*P* < .001). The FP-treated groups had slightly higher weight gain (42.7% and 41.6%) than diabetic rats untreated (37.7%) until the 6th week, but that was not statistically significant. 

 Hyperphagia and increased water intake also accompanied polyuria and glucosuria in the diabetic groups (data not shown). An elevated food intake in the diabetic rats probably would be an adaptive behavior in response to the loss of calories and cellular starvation. In response to cellular hunger for glucose in diabetes, lipolysis and proteolysis were enhanced [35]. Type 2 diabetes is caused not only by a defect in *β*-cell function and insulin resistance but also by the *α*-cell dysfunction with relative glucagon hypersecretion. Glucagon inhibits glucose-utilization pathways and the storage of metabolic fuels and also activates hepatic gluconeogenesis, glycogenolysis and lipolysis [36]. The diabetic rats had hyperphagia related to a marked hyperglycemia; consequently, glucosuria occurred along with polydypsia and osmotic diuresis in the diabetic rats. As a result of dehydration, the diabetic rats had to drink a lot of water (polydypsia) [37]. Previous studies indicated that gallic acid and rutin exhibited antihyperglycemic and antioxidative activity in STZ-induced rats [38] and had hypolipidemic effect on mice fed high fat diet [39, 40].

### 3.3. Effect of FP on Plasma Glucose Levels

**Figure 4:**
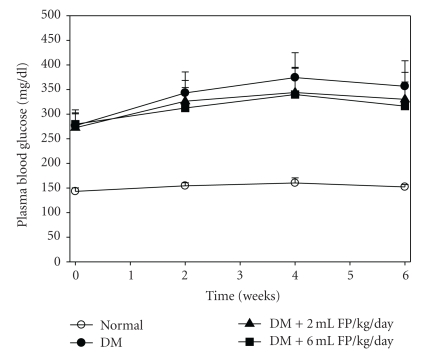
Effect of the FP on plasma glucose concentrations of STZ-induced diabetic rats. Data are expressed as mean ± SEM. ^#^ *P* < .05 when compared with the normal control group.

As shown in Figure 4, the level of plasma glucose was significantly increased (*P *<  .05) in the rats with STZ-induced diabetes (29% from 276.6 mg/dL to 356.7 mg/dL) during the entire experimental period when compared with the normal rats group (6.4% from 143.1 mg/dL to 152.2 mg/dL). However, the administration of a dose of 6 mL/kg/day FP reduced the progressive increase of plasma glucose levels during weeks 1–6 (increased only 13.1%) when compared to the diabetic control group (29%) and the low-dose group (21.1%) but not significantly. 

 Fermented fruit and plant beverages were used to improve health as well as an alternative therapy because they had anti-inflammatory and antioxidative activities [13, 18, 41]. The FP used in this study was composed of five Thai indigenous plants, rich in phenolic compounds and antioxidant activities (Table 1). The therapeutic role of FP is shown to be attributed to the improvement of diabetic oxidative stress; therefore, it may be a potential therapy for diabetes and its complications. 

The administration of FP reduced the physiological changes associated with diabetes, implying that FP normalized energy utilization and metabolism. Oxidative stress in diabetes plays a key role in vascular pathogenesis and is an early marker in development of endothelial dysfunction [42]. We found that the administration of a dose of 6 mL/kg/day of FP tended to reduce the physiological changes associated with diabetes via delaying or reducing the increase of plasma glucose level in diabetic rats better than the diabetic untreated group. This implies that high doses of FP normalized energy utilization and metabolism and possibly suppressed glucose production through the gluconeogenesis pathway. Fermentation product of blueberry is potential to modify the phenolic (chlorogenic acid and gallic acid) contents, increase antioxidant activity and increase glucose uptake by 48% in C2C12 myotubes and by 142% in 3T3-L1 adipocytes. In addition, rutin can improve hyperglycemia and dyslipidemia while inhibiting the progression of liver and heart dysfunction in STZ-induced diabetic rats.

### 3.4. Effect of FP on Plasma Lipid Peroxidation

The increased TBARS level may have an important role in pancreatic damage associated with diabetes. Increasing blood glucose levels in diabetes leads to overproduction of free radicals, defined as an imbalance between oxidants and antioxidants. Glucose autooxidizes in the presence of transition metal ions generating oxygen-free radicals, make the membrane vulnerable to oxidative damage [42–44]. Natural fermented products have been used to improve health as well as an alternative therapy, possibly due to their antioxidative, anti-inflammatory, antigenotoxic, free-radicals scavenging, and antilipid peroxidation activities [13, 17, 18, 45–48].

**Figure 5:**
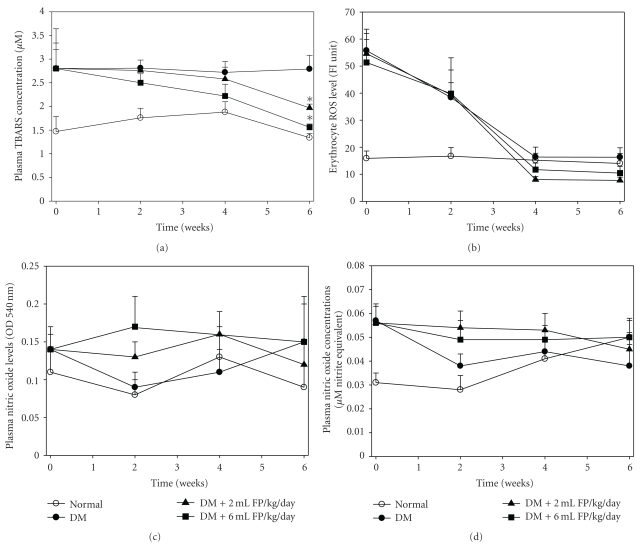
Effect of the FP on oxidative stress status of STZ-induced diabetic rats. Data are expressed as mean ± SEM. ^#^ *P* < .05 when compared with the normal control group, *P < .05 when compared with the diabetic group (DM).

Under diabetic conditions, the level of lipid peroxidation in the pancreas is much higher than in nondiabetic rats (Figure 5). During the experimental period, diabetic rats had higher lipid peroxidation in plasma than normal rats. Oxidative stress is associated with lipid peroxidation, which was analyzed by measuring TBARS levels. Lipid peroxidation is the result of a chain reaction induced by ROS and eventually leads to extensive membrane damage, dysfunction, and complications. Tissue and blood MDA levels in STZ-induced diabetic rats increased because of lipid peroxidation [49–51]. Previous studies, both *in vitro* and *in vivo*, have shown antioxidation activity to inhibit and reduce the degeneration of cells, through its antioxidation activity. Studies in Japan, Korea and Taiwan [18, 52, 53] reported that natural fermented product reduced lipid peroxidation. 

In this study, the administration of FP at doses of 2 and 6 mL/kg significantly decreased MDA levels of diabetic rats during the experimental period in a dose-dependent manner at the 6th week, when compared to the diabetic control (*P* < .05). Throughout the experiment the lipid peroxidation marker, MDA had a tendency to decrease, exhibiting a relief of oxidative stress. Obviously, FP containing phenolics (such as phenols and flavonoids) show promise as therapeutic agents for such disorders involving free radical reactions as diabetes, due to their chain-breaking antioxidants property.

### 3.5. Effect of FP on Erythrocyte Oxidative Stress

The diabetic rats had higher RBC oxidative stress than normal rats in the initial period of the experiment (Figure 5). The administration of FP resulted in a decrease (85.8% and 79.7%) in the erythrocyte ROS levels at doses of 2 and 6 mL/kg BW daily to diabetic rats from initiation to the 6th week compared with diabetic rats control group (70.8%) but not significantly. Over the experimental period, RBC oxidative stress was increased in diabetic rats, reflecting the primary of pathology of diabetic complications. Interestingly, the administration of FP for 6 weeks tended to reduce free radicals of RBC. Based on these results we would expect FP to prevent the development of diabetes and its complication. However, the data from the *in vivo *studies of FP is still limited. Beneficial health effects of phenolics depend on free-radical scavenging activities, and also due to the influence on gene expression, and the cell signaling cascades oxidative stress status.

### 3.6. Effect of FP on Plasma Superoxide and Nitric Oxide Level

As shown in Figure 5, the plasma superoxide and nitric oxide levels fluctuated during the study in all groups, except for diabetic rats treated with low-dose FP, which the levels were progressively lowered throughout the treatment, but no significant differences were found. Under diabetic conditions, hyperglycemia leads to overproduction of O_2_^•−^ and NO as a result of NAD(P)H oxidase-dependent glucose oxidation and/or nonenzymatic protein glycation. O_2_^•−^ is a mediator of endothelial dysfunction in diabetes whereas NO is responsible for harmful effects on *β*-cell function and it interacts with O_2_^•−^ to form the hydroxyl radicals, leading to highly reactive oxidative damage associated with diabetes [7, 42, 54, 55]. Our results showed that rats with STZ-induced diabetes had high plasma levels of O_2_^•−^ and NO, implying that STZ leads to oxidative stress which will ultimately affect the *β*-cell function. This study showed that FP decreased O_2_^•−^ induced by diabetes. Since the half life of O_2_^•−^ and NO are too short to measure, the effect of FP on them are uncertain. These findings suggest that FP may act as a free-radical scavenger and provide protection against the oxidative stress induced by hyperglycemia.

## 4. Conclusions

The results of this study imply that a biologically fermented product from five Thai indigenous plants including *Phyllanthus emblica, Morinda citrifolia, Houttuynia cordata, Terminalia chebula*, and *Kaempferia parviflora* markedly reduce the oxidative stress through scavenging free radicals as well as inhibiting lipid peroxidation and slightly improve glucose metabolism in STZ-induced diabetic rats. It may be a beneficial therapy or at least a delay to the pathological conditions associated with oxidative stress, which lead to diabetic complications. On the basis of this study, these findings have led to the discovery and the evaluation of antioxidative property of polyphenolics: particularly rutin, gallic acid, pyrogallol, catechin, and other phenolic compounds. Most importantly, these compounds could synergistically inhibit, at an early stage of cell damage, the mechanism leading to diabetic complications. Thai indigenous plant beverage, which is an already-available substance, should be used because their effective causal antioxidants. However, the use of FP should be further investigated for correlative and synergistic mechanisms in diabetes.

## Figures and Tables

**Table 1:** Contents of main phenolics and total antioxidant activity in the fermentation product (FP).

Measurement	Amounts
Phenolics (mg GAE/mL)	27.3 ± 1.1
Flavonoids (mg QE/mL)	2.24 ± 0.02
Hydrolyzable tannins (mg GAE/mL)	0.051 ± 0.002
Antioxidant activity (mg AE/mL)	31.31 ± 0.04

Abbreviations: GAE: gallic acid equivalents, QE: quercetin equivalents, AE: ascorbic acid equivalents. Values expressed as means ± SD (*n* = 5).

## References

[1] Ceriello A (2006). Effects of Macronutrient Excess and composition on oxidative stress: relevance to diabetes and cardiovascular disease. *Current Atherosclerosis Reports*.

[2] Haffner SM, Mykkanen L, Festa A, Burke JP, Stern MP (2000). Insulin-resistant prediabetic subjects have more atherogenic risk factors than insulin-sensitive prediabetic subjects: implications for preventing coronary heart disease during the prediabetic state. *Circulation*.

[3] Khan ZA, Chakrabarti S (2003). Growth factors in proliferative diabetic retinopathy. *Experimental Diabesity Research*.

[4] Chertow B (2004). Advances in diabetes for the millennium: vitamins and oxidant stress in diabetes and its complications CME. *Medscape General Medicine*.

[5] da Ros R, Assaloni R, Ceriello A (2004). Antioxidant therapy in diabetic complications: what is new?. *Current Vascular Pharmacology*.

[6] Monnier L, Mas E, Ginet C (2006). Activation of oxidative stress by acute glucose fluctuations compared with sustained chronic hyperglycemia in patients with type 2 diabetes. *Journal of the American Medical Association*.

[7] Evans JL, Goldfine ID, Maddux BA, Grodsky GM (2003). Are oxidative stress—activated signaling pathways mediators of insulin resistance and *β*-cell dysfunction?. *Diabetes*.

[8] Kim HY, Yokozawa T, Cho EJ, Yamabe N (2004). Protective effects of the Chinese prescription Hachimi-jio-gan against diabetic oxidative stress. *Journal of Pharmacy and Pharmacology*.

[9] Jung CH, Seog HM, Choi IW, Choi HD, Cho HY (2005). Effects of wild ginseng (Panax ginseng C.A. Meyer) leaves on lipid peroxidation levels and antioxidant enzyme activities in streptozotocin diabetic rats. *Journal of Ethnopharmacology*.

[10] El-Alfy AT, Ahmed AAE, Fatani AJ (2005). Protective effect of red grape seeds proanthocyanidins against induction of diabetes by alloxan in rats. *Pharmacological Research*.

[11] Maisuthisakul P, Gordon MH, Pongsawatmanit R, Suttajit M (2007). Enhancing the oxidative stability of rice crackers by addition of the ethanolic extract of phytochemicals from Cratoxylum formosum Dyer. *Asia Pacific Journal of Clinical Nutrition*.

[12] Huang Z, Wang B, Eaves DH, Shikany JM, Pace RD (2007). Total phenolics and antioxidant capacity of indigenous vegetables in the southeast United States: Alabama Collaboration for Cardiovascular Equality Project. *International Journal of Food Sciences and Nutrition*.

[13] Schubert SY, Lansky EP, Neeman I (1999). Antioxidant and eicosanoid enzyme inhibition properties of pomegranate seed oil and fermented juice flavonoids. *Journal of Ethnopharmacology*.

[14] Cheigh HS, Park KY (1994). Biochemical, microbiological, and nutritional aspects of kimchi (Korean fermented vegetable products). *Critical Reviews in Food Science and Nutrition*.

[15] Eriksson CE, Na A (1995). Antioxidant agents in raw materials and processed foods. *Biochemical Society Symposium*.

[16] Kroon PA, Faulds CB, Ryden P, Williamson G (1996). Solubilisation of ferulic acid from plant cell wall materials in a model human gut system. *Biochemical Society Transactions*.

[17] Minamiyama Y, Takemura S, Tsukioka T (2007). Effect of AOB, a fermented-grain food supplement, on oxidative stress in type 2 diabetic rats. *BioFactors*.

[18] Deiana M, Assunta Dessi M, Ke B (2002). The antioxidant cocktail effective microorganism X (EM-X) inhibits oxidant-induced interleukin-8 release and the peroxidation of phospholipids in vitro. *Biochemical and Biophysical Research Communications*.

[19] Alonso Garcia A, Cancho Grande B, Simal Gandara J (2004). Development of a rapid method based on solid-phase extraction and liquid chromatography with ultraviolet absorbance detection for the determination of polyphenols in alcohol-free beers. *Journal of Chromatography A*.

[20] Re R, Pellegrini N, Proteggente A, Pannala A, Yang M, Rice-Evans C (1999). Antioxidant activity applying an improved ABTS radical cation decolorization assay. *Free Radical Biology and Medicine*.

[21] Piccinelli AL, de Simone F, Passi S, Rastrelli L (2004). Phenolic constituents and antioxidant activity of Wendita calysina leaves (burrito), a folk Paraguayan tea. *Journal of Agricultural and Food Chemistry*.

[22] Vinson JA, Su X, Zubik L, Bose P (2001). Phenol antioxidant quantity and quality in foods: fruits. *Journal of Agricultural and Food Chemistry*.

[23] Kosalec I, Bakmaz M, Pepeljnjak S, Vladimir-Knezevic S (2004). Quantitative analysis of the flavonoids in raw propolis from northern Croatia. *Acta Pharmaceutica*.

[24] Hollman PCH, van Trijp JMP, Mengelers MJB, de Vries JHM, Katan MB (1997). Bioavailability of the dietary antioxidant flavonol quercetin in man. *Cancer Letters*.

[25] Polshettiwar S, Ganjiwale R, Wadher S, Yeole P (2007). Spectrophotometric estimation of total tannins in some ayurvedic eye drops. *Indian Journal of Pharmaceutical Sciences*.

[26] Blondel O, Bailbe D, Portha B (1989). In vivo insulin resistance in streptozotocin-diabetic rats—evidence for reversal following oral vanadate treatment. *Diabetologia*.

[27] Trinder P (1969). Determination of blood glucose using an oxidase-peroxidase system with a non-carcinogenic chromogen. *Journal of Clinical Pathology*.

[28] Chirico S (1994). High-performance liquid chromatography-based thiobarbituric acid tests. *Methods in Enzymology*.

[29] Amer J, Goldfarb A, Fibach E (2003). Flow cytometric measurement of reactive oxygen species production by normal and thalassaemic red blood cells. *European Journal of Haematology*.

[30] Ewing JF, Janero DR (1995). Microplate superoxide dismutase assay employing a nonenzymatic superoxide generator. *Analytical Biochemistry*.

[31] Misko TP, Schilling RJ, Salvemini D, Moore WM, Currie MG (1993). A fluorometric assay for the measurement of nitrite in biological samples. *Analytical Biochemistry*.

[32] Kusirisin W, Srichairatanakool S, Lerttrakarnnon P (2009). Antioxidative activity, polyphenolic content and anti-glycation effect of some Thai medicinal plants traditionally used in diabetic patients. *Medicinal Chemistry*.

[33] Bramati L, Aquilano F, Pietta P (2003). Unfermented rooibos tea: quantitative characterization of flavonoids by HPLC-UV and determination of the total antioxidant activity. *Journal of Agricultural and Food Chemistry*.

[34] Grun CH, van Dorsten FA, Jacobs DM (2008). GC-MS methods for metabolic profiling of microbial fermentation products of dietary polyphenols in human and in vitro intervention studies. *Journal of Chromatography B*.

[35] Ronald AA (1982). Clinical correlates of metabolic derangement of diabetes. *Clinical Diabetes Mellitus*.

[36] Unger RH (1978). Role of glucagon in the pathogenesis of diabetes: the status of the controversy. *Metabolism*.

[37] Kamtchouing P, Sokeng SD, Moundipa PF, Watcho P, Jatsa HB, Lontsi D (1998). Protective role of Anacardium occidentale extract against streptozotocin-induced diabetes in rats. *Journal of Ethnopharmacology*.

[38] Aslan M, Deliorman Orhan D, Orhan N, Sezik E, Yesilada E (2007). In vivo antidiabetic and antioxidant potential of Helichrysum plicatum ssp. plicatum capitulums in streptozotocin-induced-diabetic rats. *Journal of Ethnopharmacology*.

[39] Jang S-M, Yee S-T, Choi J (2009). Ursolic acid enhances the cellular immune system and pancreatic *β*-cell function in streptozotocin-induced diabetic mice fed a high-fat diet. *International Immunopharmacology*.

[40] Hsu CL, Wu CH, Huang SL, Yen GC (2009). Phenolic compounds rutin and o-coumaric acid ameliorate obesity induced by high-fat diet in rats. *Journal of Agricultural and Food Chemistry*.

[41] Lansky EP, Newman RA (2007). Punica granatum (pomegranate) and its potential for prevention and treatment of inflammation and cancer. *Journal of Ethnopharmacology*.

[42] Ceriello A (2003). New insights on oxidative stress and diabetic complications may lead to a "causal" antioxidant therapy. *Diabetes Care*.

[43] Baynes JW (1991). Role of oxidative stress in development of complications in diabetes. *Diabetes*.

[44] Ceriello A (2006). Oxidative stress and diabetes-associated complications. *Endocrine Practice*.

[45] Kantachote D, Charernjiratrakul W (2008). Selection of lactic acid bacteria from fermented plant beverages to use as inoculants for improving the quality of the finished product. *Pakistan Journal of Biological Sciences*.

[46] Barclay C, Mathers N (2008). Diabetes prevention. *British Journal of General Practice*.

[47] Duangjitcharoen Y, Kantachote D, Ongsakul M, Poosaran N, Chaiyasut C (2008). Selection of probiotic lactic acid bacteria isolated from fermented plant beverages. *Pakistan Journal of Biological Sciences*.

[48] Kim NY, Song EJ, Kwon DY, Kim HP, Heo MY (2008). Antioxidant and antigenotoxic activities of Korean fermented soybean. *Food and Chemical Toxicology*.

[49] Feillet-Coudray C, Rock E, Coudray C (1999). Lipid peroxidation and antioxidant status in experimental diabetes. *Clinica Chimica Acta*.

[50] Shah G, Pinnas JL, Lung CC, Mahmoud S, Mooradian AD (1994). Tissue-spectfic distribution of malondialdehyde modified proteins in diabetes mellitus. *Life Sciences*.

[51] Kakkar R, Kalra J, Mantha SV, Prasad K (1995). Lipid peroxidation and activity of antioxidant enzymes in diabetic rats. *Molecular and Cellular Biochemistry*.

[52] Chui CH, Cheng GY, Ke B (2004). Growth inhibitory potential of effective microorganism fermentation extract (EM-X) on cancer cells. *International Journal of Molecular Medicine*.

[53] Hu CC, Hsiao CH, Huang SY (2004). Antioxidant activity of fermented soybean extract. *Journal of Agricultural and Food Chemistry*.

[54] Beckman JS, Beckman TW, Chen J, Marshall PA, Freeman BA (1990). Apparent hydroxyl radical production by peroxynitrite: implications for endothelial injury from nitric oxide and superoxide. *Proceedings of the National Academy of Sciences of the United States of America*.

[55] Tesfamariam B (1994). Free radicals in diabetic endothelial cell dysfunction. *Free Radical Biology and Medicine*.

